# Oxygen and carbon isoscapes for the Baltic Sea: Testing their applicability in fish migration studies

**DOI:** 10.1002/ece3.2841

**Published:** 2017-03-06

**Authors:** Jyrki Torniainen, Anssi Lensu, Pekka J. Vuorinen, Eloni Sonninen, Marja Keinänen, Roger I. Jones, William P. Patterson, Mikko Kiljunen

**Affiliations:** ^1^ Natural History MuseumUniversity of JyvaskylaJyvaskylaFinland; ^2^ Department of Biological and Environmental ScienceUniversity of JyvaskylaJyvaskylaFinland; ^3^ Natural Resources Institute FinlandHelsinkiFinland; ^4^ Laboratory of ChronologyUniversity of HelsinkiHelsinkiFinland; ^5^ Saskatchewan Isotope LaboratoryDepartment of Geological SciencesUniversity of SaskatchewanSaskatoonSKCanada

**Keywords:** isotopic landscape, micromilling, model evaluation, *Salmo salar*, spatial assignment, spatial interpolation

## Abstract

Conventional tags applied to individuals have been used to investigate animal movement, but these methods require tagged individuals be recaptured. Maps of regional isotopic variability known as “isoscapes” offer potential for various applications in migration research without tagging wherein isotope values of tissues are compared to environmental isotope values. In this study, we present the spatial variability in oxygen ($\delta^{18}O_{H_{2}O}$) and dissolved inorganic carbon (δ^13^ C_DIC_) isotope values of Baltic Sea water. We also provide an example of how these isoscapes can reveal locations of individual animal via spatial probability surface maps, using the high‐resolution salmon otolith isotope data from salmon during their sea‐feeding phase in the Baltic Sea. A clear latitudinal and vertical gradient was found for both $\delta^{18}O_{H_{2}O}$ and δ^13^ C_DIC_ values. The difference between summer and winter in the Baltic Sea $\delta^{18}O_{H_{2}O}$ values was only slight, whereas δ^13^ C_DIC_ values exhibited substantial seasonal variability related to algal productivity. Salmon otolith δ^18^O_oto_ and δ^13^C_oto_ values showed clear differences between feeding areas and seasons. Our example demonstrates that dual isotope approach offers great potential for estimating probable fish habitats once issues in model parameterization have been resolved.

## 1 Introduction

**Figure 1:**
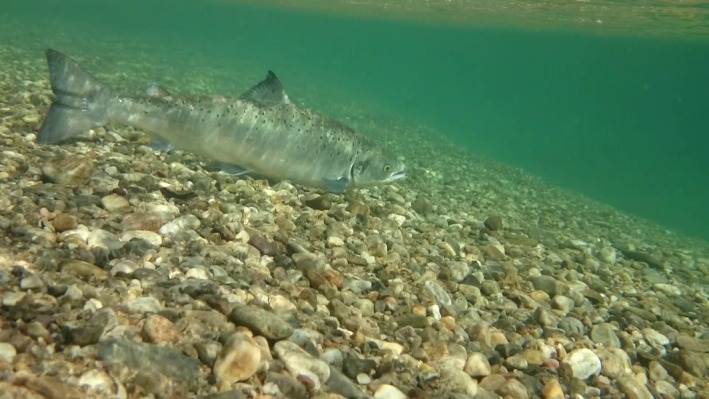
Atlantic salmon (*Salmo salar*)

Several marking approaches have been employed to address questions in migration ecology. Until recently, conventional extrinsic markers (i.e., tags) applied to individuals have been used to investigate their movement (Lucas & Baras, 2000), but these methods require tagged individuals be recaptured to acquire spatial information. During recent years some investigations have been conducted to study long‐term movements of individual adult Atlantic salmon (*Salmo salar* L.; Figure 1) in the sea using tags that record environmental characteristics along the migration routes (e.g., Chittenden, Ådlandsvik, Pedersen, Righton, & Rikardsen, 2013). However, due to the present size of the tags, the studied fish have to be large and, therefore, do not necessarily represent the majority of the population.

Intrinsic biochemical markers such as stable isotopes can provide an alternative approach to track individual movements over large geographical distances such as between continents (Hobson & Norris, 2008). All animals are isotopically marked by the environment they live in and by their diet. The assignment of an individual to a certain area works by estimating probabilities of occurrence for animal individuals by comparing values obtained from tissue samples to isotopic landscapes (i.e., isoscapes; Wunder, 2010). A great advantage in using biochemical markers is that they can be linked to those individuals that actually survived the migration to their breeding habitats, and therefore, better represent the population. As fish otoliths are almost completely mineralized from the carbonate of the environmental water (Kim, O′Neil, Hillaire‐Marcel, & Mucci, 2007; Patterson, Smith, & Lohmann, 1993; Solomon et al., 2006), analysis and comparison of otolith and water stable isotopes can reveal the locations where the otolith of an individual fish is formed. However, if the chosen tissues/materials are dissimilar, spatial assignment (matching of tissue and source isotope values) needs fractionation equations between the chosen tissue and source isotope values due to the different fractionation of the element isotopes via environmental and physiological factors.

Isoscapes offer potential for various applications in environmental and ecological research (Bowen, Wassenaar, & Hobson, 2005; Dawson & Siegwolf, 2007; Hobson & Wassenaar, 2008), whereby the isotope values of selected tissues are related to environmental isotope values, and not just one element, but several elements (i.e., multi‐isotope isoscape; e.g., Hobson et al., 2012; García‐Pérez & Hobson, 2014). The most ambitious approaches have been the constructions of isoscapes on a global scale (e.g., Amundson et al., 2003; Bowen & Revenaugh, 2003; LeGrande & Schmidt, 2006), where several studies have led to convincing results regarding animal migration among many taxa in terrestrial and aquatic environments (Best & Schell, 1996; Chamberlain, Bensch, Åkesson, & Andersson, 2000; Hanson, Wurster, EIMF, & Todd, 2010, 2013; Hobson & Wassenaar, 1996; Wassenaar & Hobson, 1998). However, although information about animal movements and spatial usage of their habitats at the intercontinental scale is important in ecological research and conservation, many crucial events occur also at smaller scales within the distribution of smaller distance migrants. Unfortunately, the availability of isoscape data for studies at a more local spatial scale appears to be sparse or the distance of the survey stations of global isoscape data may be too large for adequate local isotopic discrimination (see Bowen & Revenaugh, 2003). Therefore, additional isotopic data are needed to increase the resolution of global isoscapes to enable more precise reconstruction of animal locations and movement.

The aims of this study were (1) to provide horizontal and vertical isotopic gridded data sets (i.e., isoscapes) of oxygen ($\delta^{18}O_{H_{2}O}$) and dissolved inorganic carbon (δ^13^C_DIC_) for the water of the Baltic Sea. (2) As an example we demonstrate the potential of these isoscapes using two Atlantic salmon individuals from the River Simojoki. Combined with the isotope data from the salmon and spatial probability surface maps, we show probable locations of individual fish in various time points during their sea‐feeding migration phase in the Baltic Sea. We also demonstrate how parameterization of the models influences on location estimates.

## 2 Material and Methods

### 2.1 Sampling and isotope analyses of water

**Figure 2:**
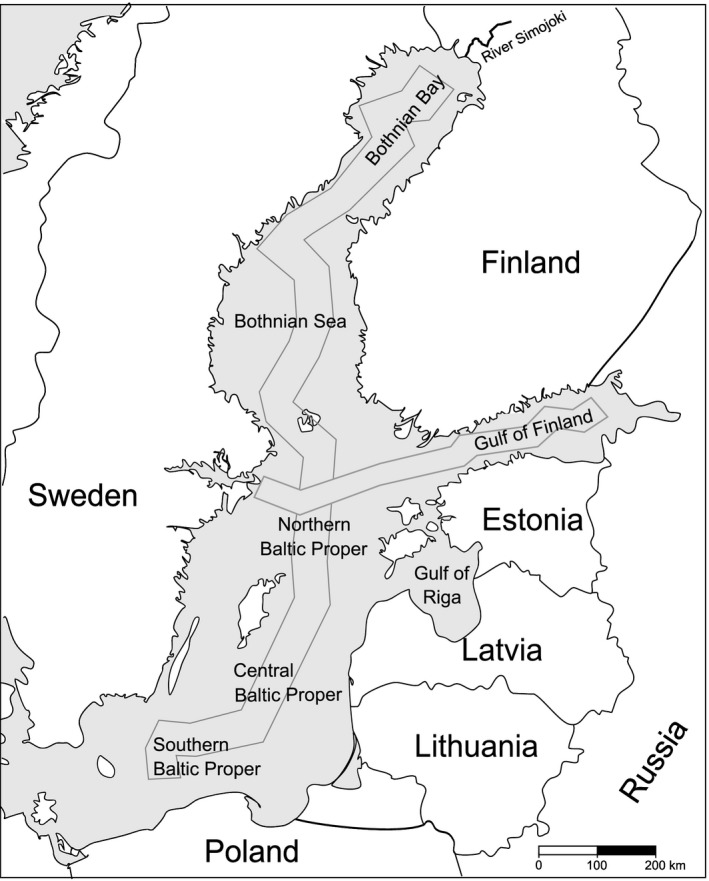
Map of the Baltic Sea with the location of the River Simojoki (uppermost right corner). The marked pathways show transects used for creating vertical interpolations in Figure 4

**Figure 3:**
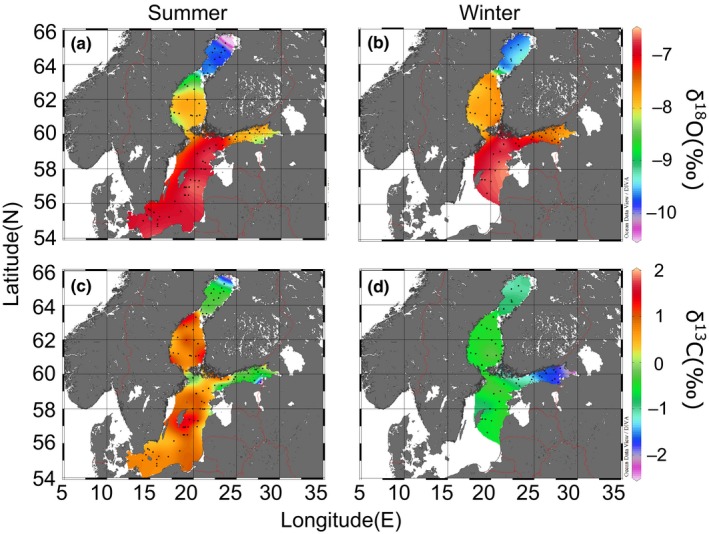
Interpolated maps for the Baltic Sea of $\delta^{18}O_{H_{2}O}$ values (a, b) and δ^13^ C_DIC_ values (c, d) from 10 meters in summer (a, c) and winter (b, d). Black dots represent sampling locations

**Figure 4:**
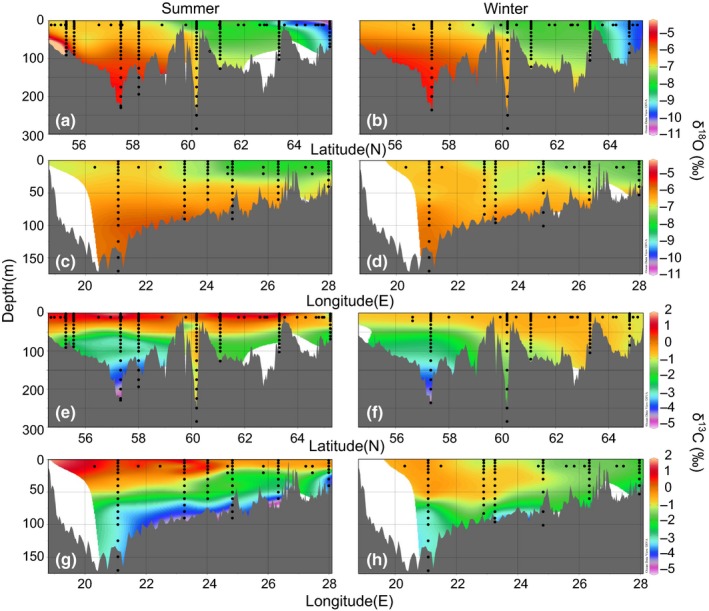
Interpolated cross‐sectional surfaces of vertical $\delta^{18}O_{H_{2}O}$ and δ^13^ C_DIC_ values. $\delta^{18}O_{H_{2}O}$ values from south to north in (a) summer and (b) winter, and from west to east in (c) summer and (d) winter. δ^13^ C_DIC_ values, from south to north in (e) summer and (f) winter, and from west to east in (g) summer and (h) winter. Black dots represent sampling locations and depths

Baltic Sea water samples were collected during three different cruises by the R/V Aranda of the Finnish Environment Institute (SYKE). To evaluate a possible seasonal impact on sea water isotope values, we collected two summer sets and one winter set of samples. The dates of the cruises were (1) 24 May to 11 June 2010, (2) 9 August to 27 August 2010, and (3) 17 January to 3 February 2011. The first cruise covered the whole Baltic Sea from the Bothnian Bay to the Southern Baltic Proper, excluding the Gulf of Finland (Figure 2), which was the only area sampled during the second cruise. The third cruise, in winter, covered the Baltic Sea except areas south of Gotland. Sea water was sampled at a depth of 10 m from every sampling station (altogether 316 samples from 134 station visits, black dots in Figures 3 and 4). From 25 stations, water was also sampled vertically at 5–50 m intervals depending on water depth at the site (Figure 4). Maximum distances between sampling stations were less than 100 km. Water samples were taken using a CTD/Rosette sampler (Rosette 1015, Seabird, SBE 911/General Oceanics, SIS: Plus 500).

Rosette sampler records conductivity, from which salinity was automatically calculated. From every sampling occasion of water for isotope analysis purpose, salinity was recorded. Sample water collected for δ^13^C_DIC_ analysis, were injected into 12‐ml borosilicate Exetainer vials (cat. no 438B; Labco Ltd., High Wycombe, UK) prepared in the laboratory, where 0.2 ml of 85% orthophosphoric acid (H_3_PO_4_) was added into each vial, which was then sealed with a cap (containing a rubber septum) and flushed and filled with a helium atmosphere. In the field 2–4 ml of sea water from each station and sample depth was injected through the rubber septum into the vial. Sample water for $\delta^{18}O_{H_{2}O}$ analysis were collected in 20‐ml glass scintillation vials and filled full ensuring no air bubbles. All samples were stored in an unlit refrigerator (+4°C) in a dark room pending laboratory analysis. In the laboratory, similar vials as used for δ^13^C_DIC_ samples were filled with 0.5 ml sea water and equilibrated with CO_2_ for at least 24 hr at 25°C. Analyses of samples started within a week after arrival to the laboratory, first the δ^13^C_DIC_ samples immediately after arrival. The δ^13^C_DIC_ values are expressed relative to VPDB (Vienna Pee Dee Belemnite), measured and calibrated/normalized against international IAEA (International Atomic Energy Agency [IAEA]) standards NBS19 (or TS‐Limestone; Calcium carbonate, δ^13^C = +1.95‰) and LSVEC (Lithium carbonate, δ^13^C = −46.6‰); ±0.14‰. Values are expressed relative to VSMOW and calibrated/normalized to the VSMOW (Vienna Standard Mean Ocean Water [VSMOW], δ^18^O = 0‰)—SLAP (Standard Light Antarctic Precipitation, δ^18^O = −55.5‰) scale; ±0.1‰. All water samples were analyzed for both δ^13^C_DIC_ and $\delta^{18}O_{H_{2}O}$ at the Laboratory of Chronology, Finnish Museum of Natural History, University of Helsinki, using a GasBench II and Delta Plus XL (Thermo Fisher Scientific, Bremen, Germany).

Statistical testing of seasonal differences in $\delta^{18}O_{H_{2}O}$ and δ^13^C_DIC_ values (paired samples *t* test) was performed using PASW Statistics 18 for Windows (SPSS Inc., Chicago, IL, USA). Interpolated isoscapes for δ^13^C_DIC_ and $\delta^{18}O_{H_{2}O}$ were constructed using Ocean Data View (ODV) software version 4.5.5 (Schlitzer, 2002, 2011).

### 2.2 Otolith sampling, micromilling and stable isotope analysis

In order to test applicability of Baltic Sea isoscapes in fish migration studies, we analyzed otoliths from two example female Atlantic salmon (hereafter Baltic salmon or salmon) originally caught, as they were returning to the River Simojoki (Figure 2) to spawn in 2008, by the Natural Resources Institute Finland (LUKE) as part of the program to monitor yolk‐sac fry mortality (M74 syndrome: e.g., Keinänen et al., 2012; original LUKE code numbers SS5447 and SS5461, hereafter FISH 1 and 2, respectively). Both salmon had spent two years feeding in the sea. The origin (wild or hatchery‐reared) and age of the salmon was determined from the scale nucleus and scale growth pattern (Hiilivirta, Ikonen, & Lappalainen, 1998). FISH 1 was of wild origin (5,300 g, 80 cm) and FISH 2 was of hatchery‐reared origin (6,300 g, 81 cm). Both sagittal otoliths were removed from the head of the salmon, cleaned in deionized water to remove any remaining organic tissue, and dried overnight at 60°C.

Both otoliths were sampled for δ^13^C and δ^18^O analysis using the custom‐built three‐dimensional computer‐controlled micromilling system in the Saskatchewan Isotope Laboratory at the University of Saskatchewan following the procedure of Wurster, Patterson, and Cheatham (1999). This system allowed 31–32 sampling paths to be followed concordant with growth banding in both otoliths. Isotope ratios of samples were determined using a Finnigan MAT 253 directly coupled to a Kiel‐IV automated carbonate preparation device (Thermo–Fisher Scientific). Accuracy and precision were monitored by routine analysis of NBS‐19 standard, yielding a standard deviation for replicate standards that was consistently less than 0.09 for both δ^13^O and δ^18^O values. All otolith isotope measurements are reported in the standard delta notation (per mil) relative to the VPDB standard as δ^18^O_oto_ and δ^13^C_oto_. From the otolith data of each salmon, three sample milling paths were selected for closer inspection, namely 1st sea winter (1SW), the following summer (2SS), and the 2nd sea winter (2SW) to assign the salmon to Baltic Sea areas for each period. During the sea migration phase, Baltic salmon experience high seasonal fluctuations in ambient temperature. These periods are clearly seen in the otoliths, as otolith carbonate with the highest δ^18^O_oto_ values is accreted during cold winter period, while the lowest δ^18^O_oto_ values represent highest temperatures in the summer (Figure 6; Wurster & Patterson, 2003).

### 2.3 Creating isotopic (isoscapes) and temperature maps for the Baltic Sea

Isoscapes for δ^13^C_DIC_ and $\delta^{18}O_{H_{2}O}$ as well as temperature maps were constructed using ODV software. Used map data for horizontal isotope maps were received from the database of the Leibniz Institute for Baltic Sea Research Warnemünde (IOW; Seifert, Tauber, & Kayser, 2001) and for both horizontal maps and vertical profiles from the General Bathymetric Chart of the Oceans (GEBCO_08 Grid). GEBCO_08 Grid had to be converted into NetCDF format compatible with ODV with R Statistics software v 3.0.1 (R Core Team, 2013) using package RNetCDF (Michna, 2012). Interpolated maps were produced using Data Interpolating Variational Analysis (DIVA) gridding software (Troupin et al., 2012) included in ODV (DIVA parameters: scale lengths chosen automatically; signal‐to‐noise ratio = 40; quality limit = 3.0; excluding outliers). Vertical profiles were also created using DIVA gridding (scale lengths chosen depending on used data; signal‐to‐noise ratio = 40; quality limit 3.0; excluding outliers). The coastlines in the maps are based on the Global Self‐consistent Hierarchical High‐resolution Shorelines database v 2.1 (Wessel & Smith, 1996).

As the spatial coverage of collected $\delta^{18}O_{H_{2}O}$ was relatively sparse for assignment models, and some larger areas lacked measurements, we created predictive models to estimate $\delta^{18}O_{H_{2}O}$ values from the Baltic Sea water salinity (S) data which is an excellent predictor for $\delta^{18}O_{H_{2}O}$ and is commonly used to estimate ocean seawater isotope values (e.g., LeGrande & Schmidt, 2006). The $\delta^{18}O_{H_{2}O}$–S relationship is frequently linearly related (LeGrande & Schmidt, 2006), but in the Baltic freshwater inflow from large rivers into Gulf of Bothnia and Gulf of Finland have an effect on the relationships. Therefore, we created separate models for the Gulf of Bothnia, the Gulf of Finland and for the rest of the remaining area of the Baltic Sea. Shape of the predictive model for each area was selected based on goodness of fit and model residuals were evaluated using quantile–quantile plots. Models were further applied to sea water salinity sampling surveys obtained from Baltic Marine Environment Protection Commission (HELCOM) database (Andersson, 2014) to create seasonal $\delta^{18}O_{H_{2}O}$ maps for the Baltic Sea. Data only for summer (July–August) and winter (February–March) for the period the study salmon had spent in the sea (2007–2008) were selected. As we found statistically significant seasonal difference in measured $\delta^{18}O_{H_{2}O}$ values, three separate $\delta^{18}O_{H_{2}O}$ maps [winter 2007 (1SW), summer 2007 (2SS) and winter 2008 (2SW)] were created to be used in assignment models. Modeled values were combined with measured values from the same season to gain better spatial coverage. Marked within‐season differences were not found between HELCOM and our own measurements based on visual validation of salinity data, obtained from depths 5–15 m, indicating that also $\delta^{18}O_{H_{2}O}$ values behave the same way.

Salmon summer temperatures were fixed at 11.5°C, calculated from preferred true 10 m staying depths of salmon in Baltic salmon data storage tag study by Westerberg, Sturlaugsson, Ikonen, and Karlsson (1999). For winter temperatures (2007–2008), we used all available temperature profiles collected from HELCOM database (Andersson, 2014) covering the whole Baltic Sea (mean temperatures from depths 5–15 m) and created an interpolated surface for the preferred 10 m staying depth. Interpolated metabolically derived bicarbonate δ^13^C_diet_ values were derived from δ^13^C values of salmon dietary species around the Baltic Sea (Appendix S3).

### 2.4 Creation of otolith‐related isoscapes from δ^18^OH2O, δ^13^C_DIC_, δ^13^C_diet_ and water temperature

Understanding the dependence of ambient water temperature and $\delta^{18}O_{H_{2}O}$ values on δ^18^O_oto_ is necessary to obtain comparable values of δ^18^O_oto_ and $\delta^{18}O_{H_{2}O}$ for spatial assignment of salmon (e.g., Patterson et al., 1993). Moreover, the δ^13^C_oto_ value is a mix of bicarbonate δ^13^C_DIC_ from ambient water and metabolically derived bicarbonate δ^13^C_diet_ (Solomon et al., 2006; Wurster & Patterson, 2003). We therefore corrected $\delta^{18}O_{H_{2}O}$ and δ^13^C_DIC_ values using literature‐derived fractionation equations, after which the corrected values are considered as “otolith isoscape” of both elements (Appendix S1). This permits a direct comparison of otolith and Baltic Sea water isotopes, thereby providing a probabilistic spatial assignment of salmon during their sea‐feeding phase.

Initially, $\delta^{18}O_{H_{2}O}$ values are calculated relative to the VSMOW scale and δ^18^O_oto_ values relative to the VPDB scale from the IAEA. To enable direct comparison of δ^18^O_oto(VPDB)_ with δ^18^O_water(VSMOW)_, δ^18^O_water(VSMOW)_ values were converted to VPDB using following equation (from Clark & Fritz, 1997):

$$\delta^{18}O_{H_{2}O\left(\mathrm{VPDB}\right)}=0.97002\times \delta^{18}O_{H_{2}O\left(\mathrm{VSMOW}\right)}-29.98.$$

Oxygen isotope values of otoliths reflect those of the ambient water (Campana, 1999; Farrell & Campana, 1996; Thorrold, Jones, & Campana, 1997), with a temperature‐dependent fractionation (e.g., Patterson et al., 1993). Following common practice, we used the linear temperature‐dependent fractionation (e.g., Patterson et al., 1993).

1,000×lnα=a+b/T,

where *T* is temperature (10^3^/K), where *K* is ambient water temperature in Kelvin, and parameter α is the fractionation factor between otolith and ambient water [α = (δ^18^O_oto(VPDB)_ + 1,000)/($\delta^{18}O_{H_{2}O}$_(VPDB)_ + 1,000)]. Model fractionation constants *a* and *b* in four previous studies covering salmonid fishes are variable (*a* = −41.14, *b* = 20.43: Godiksen et al., 2010; *a* = −33.43, *b* = 17.88: Hanson et al., 2013; *a* = −33.49, *b* = 18.56: Patterson et al., 1993 and *a* = −41.69, *b* = 20.69: Storm‐Suke, Dempson, Reist, & Power, 2007). To evaluate the effect and variation of different parameters to salmon assignment, the numerically most distant parameters from each other (i.e., Hanson et al., 2013; Patterson et al., 1993) were selected for salmon assignment (i.e., for probability surfaces), hereafter Model 3 and Model 1, respectively. Also an average of all four models (*a = *−37.44, *b = *19.39) was calculated and used accordingly, hereafter Model 2 (Appendix S2).

The following equation gives the otolith‐related δ^18^O_(VPDB)_ isoscape values in relation to water temperature (*T*) and $\delta^{18}O_{H_{2}O}$_(VPDB)_ values, that can be compared to δ^18^O_oto_ values enabling the assignment of salmon to sea areas based on their otolith isotope values:

$$\delta^{18}O_{\left(\mathrm{VPDB}\right)}=e^{(a+b/T)/1,000}\times \left(\delta^{18}O_{H_{2}O\left(\mathrm{VPDB}\right)}+1,000\right)-1,000.$$

An otolith‐related δ^13^C_oto_ isoscape was calculated as follows (e.g., Solomon et al., 2006; Wurster & Patterson, 2003) to allow comparison between δ^13^C_DIC_ and δ^13^C_oto_:

$$\delta^{13}C_{\mathrm{oto}}=M\times \delta^{13}C_{\mathrm{diet}}+\left(1-M\right)\times \delta^{13}C_{\mathrm{DIC}},$$

where δ^13^C_diet_ is a mean δ^13^C value of salmon primary prey species in the Baltic Sea (sprat [*Sprattus sprattus*], Baltic herring [*Clupea harengus membras*] and three‐spined stickleback [*Gasterosteus aculeatus*]) in a particular location (Appendix S3) and M is the proportion of metabolic carbon in the salmon otolith (Sherwood & Rose, 2003):

M=0.025+0.066×Kcaud,

where *Kcaud* (Atlantic salmon caudal fin ratio; Minns, King, & Portt, 1993) is 2.4.

### 2.5 Salmon assignment using otolith and water isotope values

To estimate the locations of salmon individuals in their 1SW, 2SS and second 2SW from δ^18^O_oto_ and δ^13^C_oto_ values, we calculated probability density surfaces for each salmon by using a deterministic grid covering the Baltic Sea following the approach presented in Wunder (2010) with R Statistics software v 3.0.1 (R Core Team 2013). We first had to reinterpolate all DIVA interpolation results (water and diet‐based isotopes, temperatures, etc. which were in different kinds of nondeterministic grids) from ODV into a deterministic grid to enable us to calculate the fractionation equations for all grid locations. This reinterpolation was performed with local (*N*_max_ **= 4) inverse‐distance weighting interpolation available in the R package gstat (Pebesma, 2004). The probability density surface calculation approach assumes water‐temperature‐dependent δ^18^O_(VPDB)_ values and DIC‐diet‐based δ^13^C values to be normally distributed at each grid point and then calculates the probabilities of obtaining the measured values of the otolith from normal distributions with mean parameters according to the VPDB‐corrected interpolated isotope values. Standard deviations of δ^18^O and δ^13^C values were estimated based on observed variations of $\delta^{18}O_{H_{2}O}$ (ɛ = 0.019) and δ^13^C_DIC_ (ɛ = 0.216) values obtained from the same location and depth, and the variation of observed δ^18^O_oto_ values in the otoliths of several fish individuals (ɛ = 0.207 estimated from Godiksen et al., 2010).

The resulting probability surfaces do not represent two‐dimensional probability surfaces (total probability in each maps is not scaled to be 1) and the minimum and maximum probability values are not the same in all maps as is common in this kind of approach (see e.g., Wunder, 2010). Instead, these surfaces visualize where the probability of obtaining the measured value of the otolith (or the probability of presence of particular individual at a certain time) is the greatest, and where it is (much) lower. In those maps where both oxygen and carbon isotopes have been taken into account, probabilities were calculated by multiplying isotope‐wise probabilities, assuming independence, which may not be strictly true for this kind of phenomenon. However, our data did not allow for full estimation of covariance between true data values due to partially differing measurement or observation locations. Nevertheless, the covariance of interpolated surfaces was about 10‐fold smaller than the variances of individual isotopes. Therefore, we expect that any possible error in the results, due to dependence in isotope values, is small.

To study, how sensitive our approach is to measurement errors related to the stable isotopes, we conducted a simple sensitivity analysis by adding and/or subtracting the observed standard deviations of water samples (based on literature) of δ^13^C and δ^18^O to/from the actual measured values, and performed the calculation of the probability of presence for FISH 2 on the first winter (1SW) using the model by Hanson et al. (2013) for fractionation. The amount ±*SD* of water isotope measurement variation (based on literature) was chosen because it also reflects about ±2 *SD* of our own estimate of measurement accuracy (0.09) of otolith isotope values. So, these limits are also rather close to being about 95% confidence interval of the measured isotope values.

## 3 Results

### 3.1 δ^18^OH2O, and δ^13^C_DIC_ values in the Baltic Sea

Difference in $\delta^{18}O_{H_{2}O}$ values between seasons at 10 m depth at the same locations was statistically significant (Paired samples *T* test: *N *=* *31, *p *<* *.001). However, the mean (±*SD*) values of $\delta^{18}O_{H_{2}O}$ were very similar (mean_summer_: −7.9‰ ± 0.83, mean_winter_: −7.6‰ ± 0.74). In addition, $\delta^{18}O_{H_{2}O}$ values showed a clear south to north latitudinal decrease from around −6‰ to around −10‰ (Figure 3a,b). A weaker longitudinal decrease from around −6‰ to around −7.5‰ occurred from the eastern end of the Gulf of Finland to the western side of the Northern Baltic Proper (Figure 3a,b). Distinct differences in $\delta^{18}O_{H_{2}O}$ values between adjacent basins were also observed (Bothnian Bay—Bothnian Sea—Baltic Proper—Gulf of Finland), but the Bothnian Sea and the Gulf of Finland, even though not directly connected, had very similar values (Figure 3a,b) due to the influx of fresh water from rivers.

Mean (±*SD*) δ^13^C_DIC_ values were significantly different (Paired samples *T* test: *N *=* *31, *p *<* *.001) between sampling times (mean_summer_: 0.3‰ ± 0.81, mean_winter_: −0.9‰ ± 0.54). δ^13^C_DIC_ values decreased with latitude in summer from around 2‰ to around −2‰ (Figure 3c). The difference was smaller in the winter (Figure 3d). An increasing trend was observed in δ^13^C_DIC_ values from the Gulf of Finland to the Baltic Proper in both seasons, from around −1‰ to around 2‰ in summer and from around −2‰ to around 0‰ in winter (Figure 3c,d).

Vertical interpolation of the transects showed that $\delta^{18}O_{H_{2}O}$ values in the Central Baltic Proper were higher below ~50 m from around −6.5‰ to around −5.5‰ at the bottom and as high as −4‰ in the Southern Baltic Proper (Figure 4a,b), whereas a clear decreasing trend in summer δ^13^C_DIC_ values from around −1‰ to around −5‰ at the bottom was observed (Figure 4e,f). Vertical interpolation also showed stability of $\delta^{18}O_{H_{2}O}$ values between summer and winter, whereas δ^13^C_DIC_ values exhibited variation between summer and winter in the upper water layer (up from ~50 m; Figure 4e–h). Below ~50 m both values were rather stable over time, except in the less saline areas (the Bothnian Sea, the Bothnian Bay and the Gulf of Finland) where δ^13^C_DIC_ values showed minor decreases due to autumn water column turnover (Figure 4).

**Figure 5:**
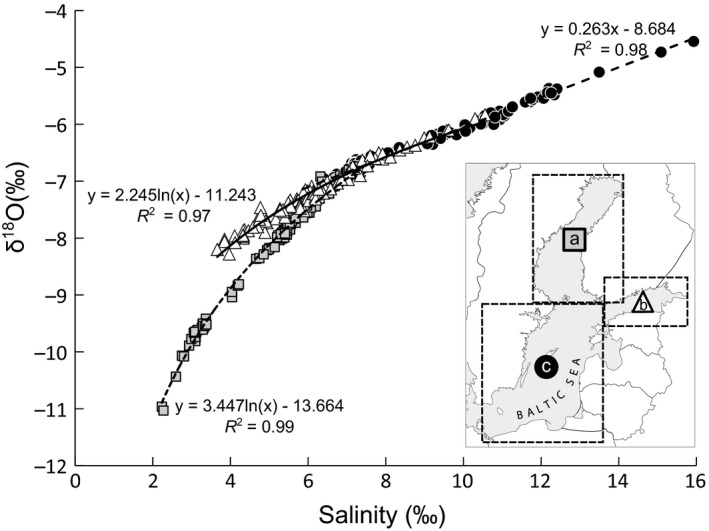
Relationships between sea water salinity and $\delta^{18}O_{H_{2}O}$ measured in (a) Gulf of Bothnia, (b) Gulf of Finland and (c) Baltic Proper. Presented models were applied when estimating $\delta^{18}O_{H_{2}O}$ for each area

There was a strong $\delta^{18}O_{H_{2}O}$–S relationship and the coefficients of determination for all the models were close to 1. Relationship appeared to be linear only for Southern Baltic Sea (*R* ^2^ = .98), while in Gulf of Finland (*R* ^2^ = .97) and Gulf of Bothnia relationships where of logarithmic shape (*R* ^2^ = .99; Figure 5).

### 3.2 Carbon and oxygen isotope values of salmon otoliths

**Figure 6:**
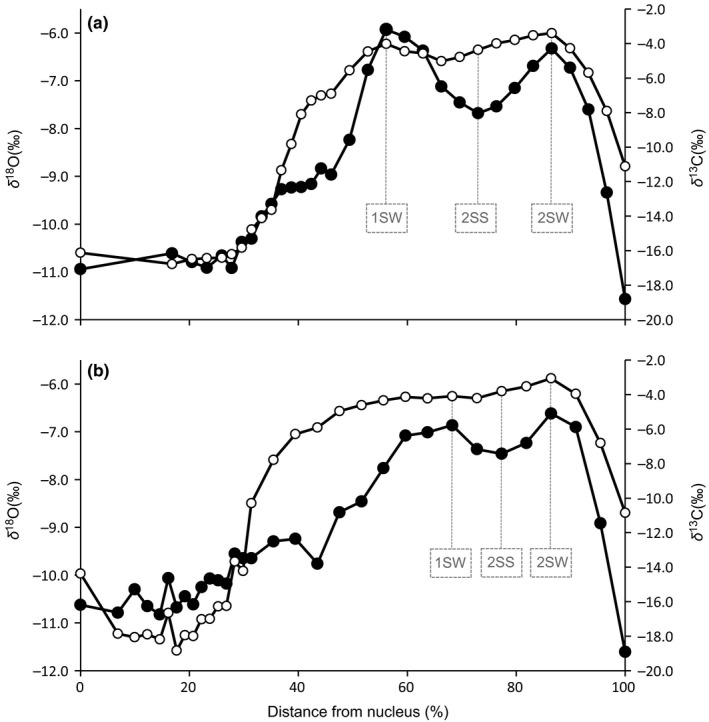
Isotope values of two example River Simojoki salmon otoliths micro‐milled and analyzed for oxygen and carbon stable isotopes: (a) FISH 1 (wild origin) and (b) FISH 2 (hatchery‐reared). Open circles represent δ^13^C values and filled circles δ^18^O values. Isotope point values used in the salmon assignment to the probable locations in the Baltic Sea during their first (1SW) and second sea winter (2SW) and second sea summer (2SS) are indicated

Stable isotope analysis via micromilling from the otolith nucleus to the otolith edge showed clear variation in otolith isotope values. In addition, both isotope values of the two otoliths analyzed showed similar life‐history trends from the nucleus to the otolith edge. δ^18^O values in the otolith nucleus (FISH 1: −10.9‰; FISH 2: −10.6‰) were markedly lower than the highest values observed in the outer part of the otolith radius (FISH 1: −5.9‰; FISH 2: −6.6‰), and with the lowest values at the edge of the otolith (both otoliths: −11.6‰; Figure 6a,b).

For both otoliths, δ^13^C values also showed clear variation along the otolith radius, from lowest δ^13^C values in the otolith nucleus (FISH 1: −16.1‰; FISH 2: −14.4‰) to highest in the outer part of the otolith radius (FISH 1: −3.4‰, FISH 2: −3.1‰), with intermediate values at the edge of the otolith (FISH 1: −11.1‰; FISH 2: −10.8‰; Figure 6a,b). Both otolith nuclei (i.e., during the juvenile phase) exhibited low δ^13^C_oto_ and δ^18^O_oto_ values. The juvenile phase isotope values of FISH 1 tended to be less variable than those of FISH 2. The otolith isotope values of apparent post‐smolt migration from the fresh water to more saline are the most distinct between these two salmon. FISH 1 otolith δ^18^O values increased to the highest values more sharply than those of FISH 2, for which δ^13^C values increased in a more linear manner. Otolith δ^18^O values for FISH 1 were higher during the first sea winter than those for FISH 2, while values during the second sea winter and apparent spawning migration (steep decline in both isotope values after 2SW) were similar for both salmon (Figure 6a,b).

### 3.3 Probability surfaces for salmon locations in the sea‐feeding phase

**Figure 7:**
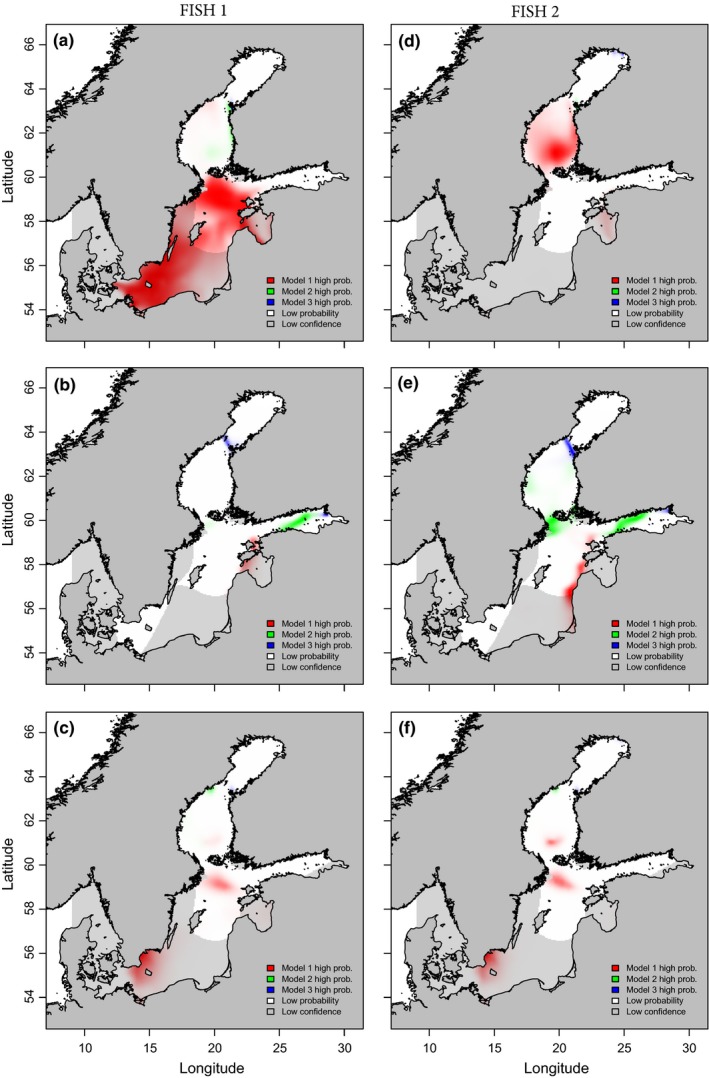
Probability surfaces of locations for two example River Simojoki Atlantic salmons (FISH 1 and 2) during their feeding phase of first sea winter (1SW; a and d, respectively), second sea summer (2SS; b and e, respectively) and second sea winter (2SW; c and f, respectively) in the Baltic Sea. Calculations are based on salmon otolith and Baltic Sea water δ^18^O and δ^13^C values. White and high‐saturation colors indicate that all value surfaces used in the calculation of probabilities (temperature, $\delta^{18}O_{H_{2}O}$, δ^13^ C_DIC_ and prey isotope values) are reliable in contrast to low confidence probabilities indicated in gray and grayish colors. Probability surface results indicated in red represent high probability of presence according to the used model of Hanson et al. (2013) (Model 1), green color indicates high probability or presence with the average model of all four used models in this study (Model 2; Godiksen et al., 2010; Hanson et al., 2013; Patterson et al., 1993; Storm‐Suke et al., 2007) and blue color represents high probability or presence on the model probabilities of Patterson et al. (1993) (Model 3)

Based on the Model 1 (Hanson et al., 2013) assignments, the most probable first sea winter location of FISH 1 was in the Baltic Proper (Figure 7a). During the next summer and the second winter, FISH 1 appeared to occupy waters close to the Gulf of Riga, and the areas of the southwest and northern Baltic Proper, respectively (Figure 7b,c). The results for FISH 2 were very similar, except that in the first winter it appeared to be located in the Bothnian Sea or in the Gulf of Riga (Figure 7d–f). Compared to the Model 1, assignments of the Model 3 (Patterson et al., 1993) were located more northern and also colder areas or to areas with lower salinity (the Gulf of Finland; Figure 7). Model 2 (the average model of four models used in the study) salmon assignments were between Hanson et al. (2013) and Patterson et al. (1993) models (Figure 7). Some of the assignments of the models 2 and 3 were confined to such a small area that results of the models were not very visible in the assignment maps (Figure 7).

**Figure 8:**
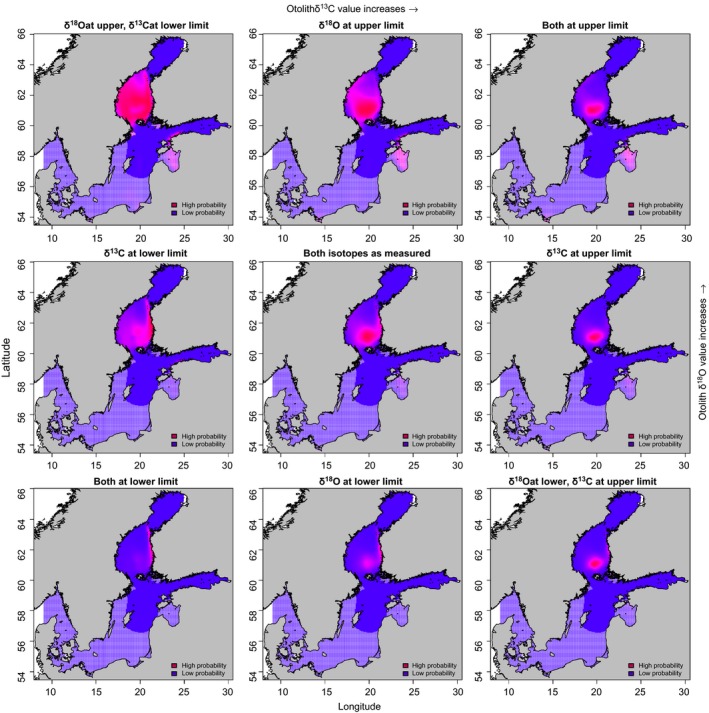
Sensitivity analysis of probabilistic spatial assignment Model 1 for FISH 2 in 1st winter at the sea (1SW). Original otolith δ^18^O and δ^13^C values (figure in the middle) were adjusted ±1*SD*. High probability means where the probability of obtaining the measured (or deviated) value of the otolith (or the probability of presence of this individual at 1SW) from the created otolith isoscape is the greatest by the used model. The hatched (low‐saturation colored) areas indicate regions where the density of real observations of at least one isoscape (used in the calculation of probability of presence) is too low for reliable prediction

Total of eight different results (Figure 8) were obtained from the sensitivity analysis in addition to the result obtained with actual measured values. Increasing the otolith δ^18^O value causes the most probable area in the Gulf of Bothnia to shift slightly toward west, and decreasing it causes the most probable area to shift slightly toward east. Increasing δ^13^C value seems to make the assignment more concentrated at one location and decreasing it causes the result to be less determined.

## 4 Discussion

### 4.1 Isotope values of the Baltic Sea

We presented the distribution of δ^13^C_DIC_ and $\delta^{18}O_{H_{2}O}$ values in the Baltic Sea during both summer and winter via interpolated horizontal isotopic maps from a depth of 10 m and also via vertical cross‐sections. Differences between sea areas in both isotope values were observed. Horizontal δ^13^C_DIC_ values clearly differed between summer and winter, whereas $\delta^{18}O_{H_{2}O}$ values remained rather similar. Vertical $\delta^{18}O_{H_{2}O}$ values remained stable between summer and winter, whereas δ^13^C_DIC_ showed marked differences between summer and winter, especially near the surface.

The distinctive hydrologic characteristics of the Baltic Sea are clearly reflected in our measurements. $\delta^{18}O_{H_{2}O}$ values in the Baltic Sea are mainly controlled by the influx from the Danish straits of denser, more saline water with higher $\delta^{18}O_{H_{2}O}$ (see e.g., Dickson, 1973; Matthäus & Lass, 1995) and the fresh water with lower $\delta^{18}O_{H_{2}O}$ values running off from the catchment area. The observed vertical gradient in isotope values is also due to the penetration of more saline water with higher $\delta^{18}O_{H_{2}O}$ values under the less dense fresh water with lower $\delta^{18}O_{H_{2}O}$ values. This denser water stagnates in the deeper basins isolated by the sills between different Baltic Sea areas (Matthäus et al., 2008) and leads to the areal segregation of $\delta^{18}O_{H_{2}O}$ values. A similar phenomenon can be seen in marine environments around the world (Schmidt, Bigg, & Rohling, 1999) and has been recorded from the Southern Baltic Sea (Frohlich, Grabczak, & Rozanski, 1988; Punning, Vaikmae, & Maekvi, 1991). Temperature‐related autumn overturn of the water column breaks down the summer stratification and mixes the water above the halocline (down to ~50 m), but the deeper water column remains rather stable maintaining more marine characteristics (Lass & Matthäus, 2008). However, in our study, $\delta^{18}O_{H_{2}O}$ values remained stable between summer and winter, and only occasional strong salt water pulses (e.g., Dickson, 1973; Matthäus & Lass, 1995) could change this situation. Lack of winter isotope measurements south from Gotland might have affected the interpolated values denoted in the maps with grayish colors). The Finnish Environment Institute (SYKE; http://www.itameriportaali.fi/en_GB/: 2.6.2014) and HELCOM (Andersson, 2014) have reported stable hydrology and salinity of the Baltic during the studied years. Based on those attributes, it is not very likely that $\delta^{18}O_{H_{2}O}$ values in summer would be very different from the values observed in winter.

δ^13^C_DIC_ values are controlled by similar mechanisms as those that control the water column characteristics and $\delta^{18}O_{H_{2}O}$. The clearest distinction between the mechanisms based on temperature‐related bioactivity. Preferential incorporation of ^12^C during photosynthesis (Fry, 2006) was reflected in the distinct differences between summer and winter in the surface water values of δ^13^C_DIC_. This mechanism also offers a possibility to reveal animal movements (MacKenzie et al., 2011; Trueman, MacKenzie, & Palmer, 2012). The dark, hypoxic halocline (eventually anoxic in the very deep water) in the Central Baltic Proper and Gulf of Finland (Lass & Matthäus, 2008) constrains bioactivity and is one main reason for the lower δ^13^C_DIC_ values in the deep water compared to the upper water layer. Lowest δ^13^C_DIC_ values in the deepest water are likely due to respiration of organic matter supplying DIC to the base of the water column. The effect of lack of the southernmost winter measurements on δ^13^C_DIC_ values depends mostly on temperature, which might be slightly higher in the areas south from Gotland and therefore might have a slight positive effect on southern δ^13^C_DIC_ values.

In the oceans, the $\delta^{18}O_{H_{2}O}$–S relationship is frequently linear (LeGrande & Schmidt, 2006), but our data suggests nonlinear relationships for Gulf of Bothnia and Gulf of Finland. This is most likely due to strong freshwater input from large rivers in these areas. Nevertheless, using the shown models, $\delta^{18}O_{H_{2}O}$ values of Baltic Sea water can be predicted with surveyed salinity data. As such, modeled values clarify the Baltic Sea $\delta^{18}O_{H_{2}O}$ distribution, and also make the interpolation more reliable and raise the confidence of salmon assignment probability throughout the Baltic Sea.

### 4.2 Isotope values of otoliths and the location estimates of salmon during their sea‐feeding phase

Both otoliths’ isotope values showed clear and similar variations from the nucleus to the otolith edge for the two example salmon individuals in this study. Our results are consistent with measurements for salmon in the Atlantic environment (Hanson et al., 2010, 2013), the Pacific (Zazzo, Smith, Patterson, & Dufour, 2006) and even in early salmon‐like fish 172 million years ago in the proto‐Atlantic during the Jurassic Period (Patterson, 1999); the similar isotope values in the otolith nuclei and the outermost edges clearly show the hatching and spawning times of the individual salmon in the river. Values between the nucleus and the edge indicate the salmon life cycle from a parr in the river, through the following juvenile post‐smolt phase to the final marine‐feeding phase. Low δ^18^O and δ^13^C values in the nucleus and shortly after indicate the juvenile riverine phase of salmon (e.g., Zazzo et al., 2006). Rapid increases in both δ^18^O and δ^13^C isotope values indicated the beginning of the salmon post‐smolt migration phase continuing with the first and second sea winter feeding phases in the Baltic Sea. Temperature‐related fractionation is clearly observed between the sea winters by marked decreases in δ^18^O values. After two sea winters both isotope values decreased very rapidly, clearly recording the beginning of the spawning migration back to the natal river and its rather short duration.

Our example FISH 1 values suggest that this individual had moved into sea water more rapidly than FISH 2. Although the highest values for both salmon were nearly equal, it appears that FISH 1 occupied slightly cooler or more saline water with higher $\delta^{18}O_{H_{2}O}$ values in the winters. The missing δ^13^C measurements from the southern areas may have an effect on the assignment. However, as the Baltic Sea hydrology has remained stable the effect of missing values is likely to be small. The assignment of location does not seem to change radically in those areas where all interpolation surfaces used in the assignment have high confidence. Although differences in stable isotope composition of water between basins was not large, sensitivity analysis indicate that Model 1 was relatively robust for slight changes in otolith isotope composition. Probable locations for tested 1SW FISH 2 remained in Bothnian Sea although we made realistic modifications in isotope composition of otolith.

Temperature–fractionation relationships (Godiksen et al., 2010; Hanson et al., 2013; Patterson et al., 1993; Storm‐Suke et al., 2007) between δ^18^O_oto_ and $\delta^{18}O_{H_{2}O}$ values provide means for evaluating fish migratory pathways. Probability surfaces clearly show that the decision of the model is crucial for the assignment. In some studies, taxonomical relatedness has been used as a justification to select temperature–fractionation relationships between water and fish otoliths (Godiksen et al., 2010). In our case, models determined for different fish species provided highly variable results. Some authors have argued that model selection should be based on genus‐specific parameters (Storm‐Suke et al., 2007), but, for example, Godiksen et al. (2010) strongly recommended that selection of model parameters should be done on the basis of physiological similarity. In our examples Model 1 (Hanson et al., 2013) placed both salmon in more southern areas compared to the other two models. In contrast, Model 3 (Patterson et al., 1993) suggests salmon occupy more northern areas and Model 2 (the average version of models) set salmon somewhere between those two extremes. In addition, Model 3 probability surfaces are vague, narrow and sometimes even invisible, indicating that all parameters are not valid for the Baltic Sea. In some cases this is also true for Model 2, especially in second winter. Experimentally determined parameters for salmon do not exist, but recently presented Model 1 (Hanson et al., 2013), based on literature‐derived fractionation constant and assumptions on water temperature, is the only model developed for salmon and seemed to provide the most realistic assignments for our example individuals. Previous studies conducted using different methods (Aro, 1989; Jutila, 2008; Kallio‐Nyberg et al. 2011; Salminen, Kuikka, & Erkamo, 1994; Torniainen et al., 2014) have indicated that majority of River Simojoki salmon migrate to the Baltic Proper area for feeding. However, smaller but appreciable proportion feed in the Bothnian Sea. Based on those findings, we see the Model 1 provides most sensible results.

The Baltic Sea temperatures are highly variable between seasons and markedly higher during summer than in the Atlantic. This could be seen in the otolith δ^18^O value profiles via the clear decrease in the values between winters (e.g., Hanson et al., 2010). Summer temperatures used in our assignment method are based on Westerberg et al. (1999) study conducted using data storage tags. Therefore, the salmon preferred summer temperature should be realistic. In winter, temperatures are average values of measured temperatures from the assumed depth range occupied by salmon. The exact temperature range occupied by wild salmon in the winter in the Baltic Sea is unknown, but it has been shown that the interannual differences in winter (February/March) sea surface temperature (SST) are small, average temperature being ~0.5°C and ranging roughly between 1.5 to −0.5°C (Siegel, Gerth, & Tschersich, 2008) In addition, temperature does not change much until unsuitable hypoxic halocline for salmon is reached in winter (Lass & Matthäus, 2008).

Carbon isotope values have been shown to offer potential for salmon assignment in the Atlantic (MacKenzie et al., 2011). Therefore, we tested whether δ^13^C_oto_ and δ^13^C_DIC_ values could differentiate especially between the Gulf of Finland and the Bothnian Sea, which is difficult from oxygen isotopes alone. Substantial part (~20%) of the otolith δ^13^C is derived from diet (Solomon et al., 2006) and knowledge about the spatial variability of salmon diet δ^13^C in the Baltic is still insufficient. Therefore, possibly false assignments are at least partly due to missing δ^13^C values for prey species covering the whole Baltic Sea area. In general, our assignment models have uncertainty due to lack of present knowledge of the exact parameters for Baltic salmon and therefore there is urgent need for species‐specific experimental studies to better understand temperature–fractionation relationships and ambient environment of Baltic salmon. Such information could be obtained by conducting controlled experiments of temperature–fractionation relationships between water and salmon otoliths. Regardless of these small shortcomings, our examples illustrate the great potential of the isoscape method used in this and other studies (Correia, Barros, & Sial, 2011; Dufour, Höök, Patterson, & Rutherford, 2008; Hanson et al., 2013) for revealing fish movements during their sea‐feeding phase which are difficult to study using other methods.

In conclusion, we emphasize that δ^18^O_oto_ and δ^13^C_oto_ values offer great possibility to show habitats and estimate migration pathways of salmon and other fish species alongside other methods (Chittenden et al., 2013; Healey et al., 2000; Lucas & Baras, 2000; Peterson, Morgan, Fisher, & Casillas, 2010), but uncertainties in the set parameters need to be better resolved. In addition, we recommend that all the data (e.g., prey species, water isotope values and water temperature) used in the assignment model and the temperature–fractionation corrections should cover the whole study area to achieve the most reliable area assignments.

## Conflict of Interest

None declared.

## Data Accessibility

We provide the measured $\delta^{18}O_{H_{2}O}$ and δ^13^C_DIC_ values from the Baltic Sea and salmon otoliths for any scientist to use in the ISOBANK repository https://github.com/BrianHayden/IsoBank. R codes can be found in Dryad Digital Repository.
