# Supplementary material for: Oxygen and carbon isoscapes for the Baltic Sea: Testing their applicability in fish migration studies
Source: Ecol Evol. 2017 Mar 6;7(7):2255–67. doi: 10.1002/ece3.2841 (PMC5383481; doi:10.1002/ece3.2841)
Supplement: Supplementary file 1 [file ECE3-7-2255-s001.docx]

**
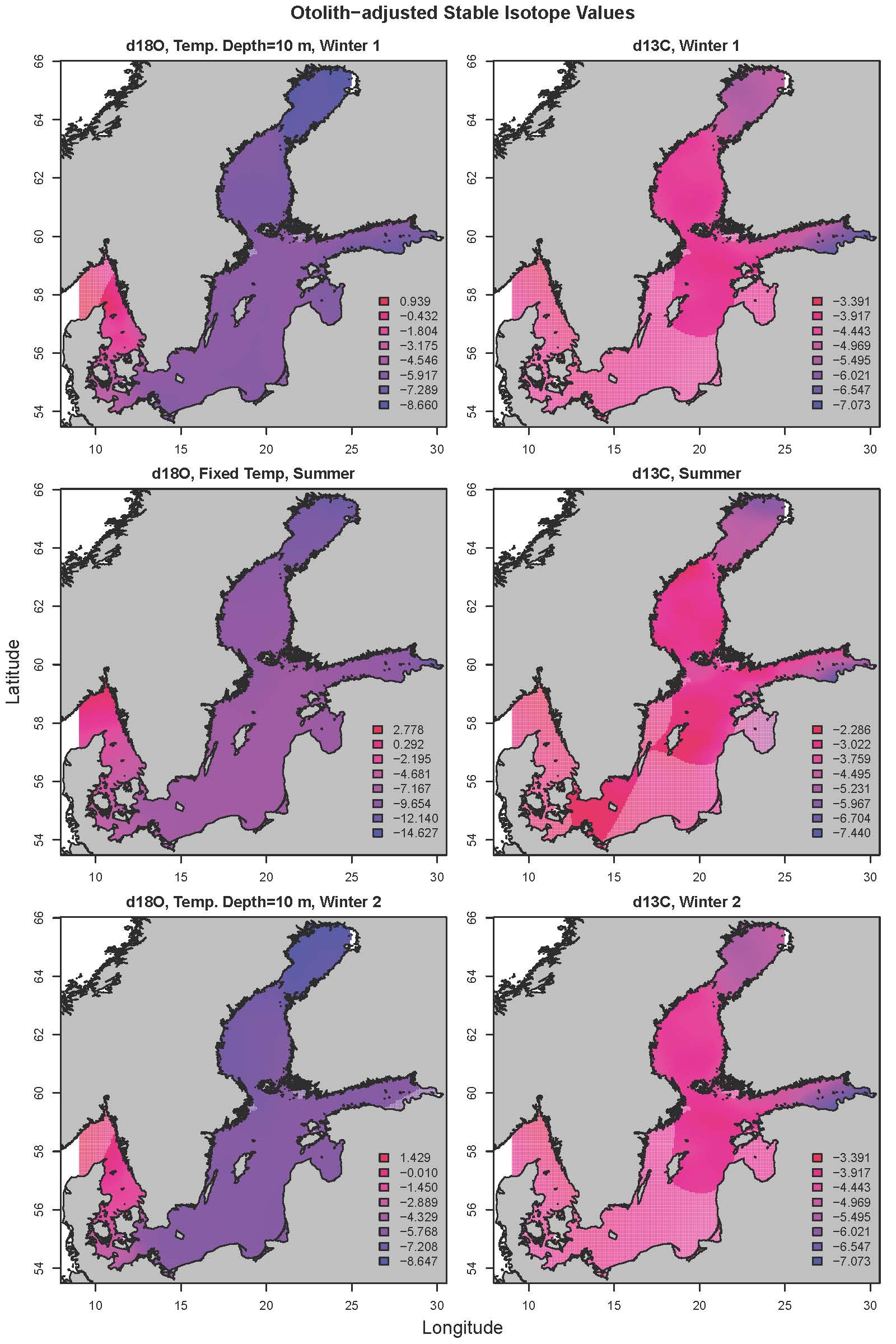
**

**Appendix S1.** Baltic salmon otolith-adjusted δ^18^O and δ^13^C isoscapes for three modelling periods [Winter1 (1SW), summer (2SS) and Winter2 (2SW)] based on fractionation equations in Model 1. Isoscapes were used for probabilistic spatial assignment of salmon during their sea-feeding phase. Greyish colours indicate areas of low confidence (where the interpolation may not be reliable due to sparse data).

**
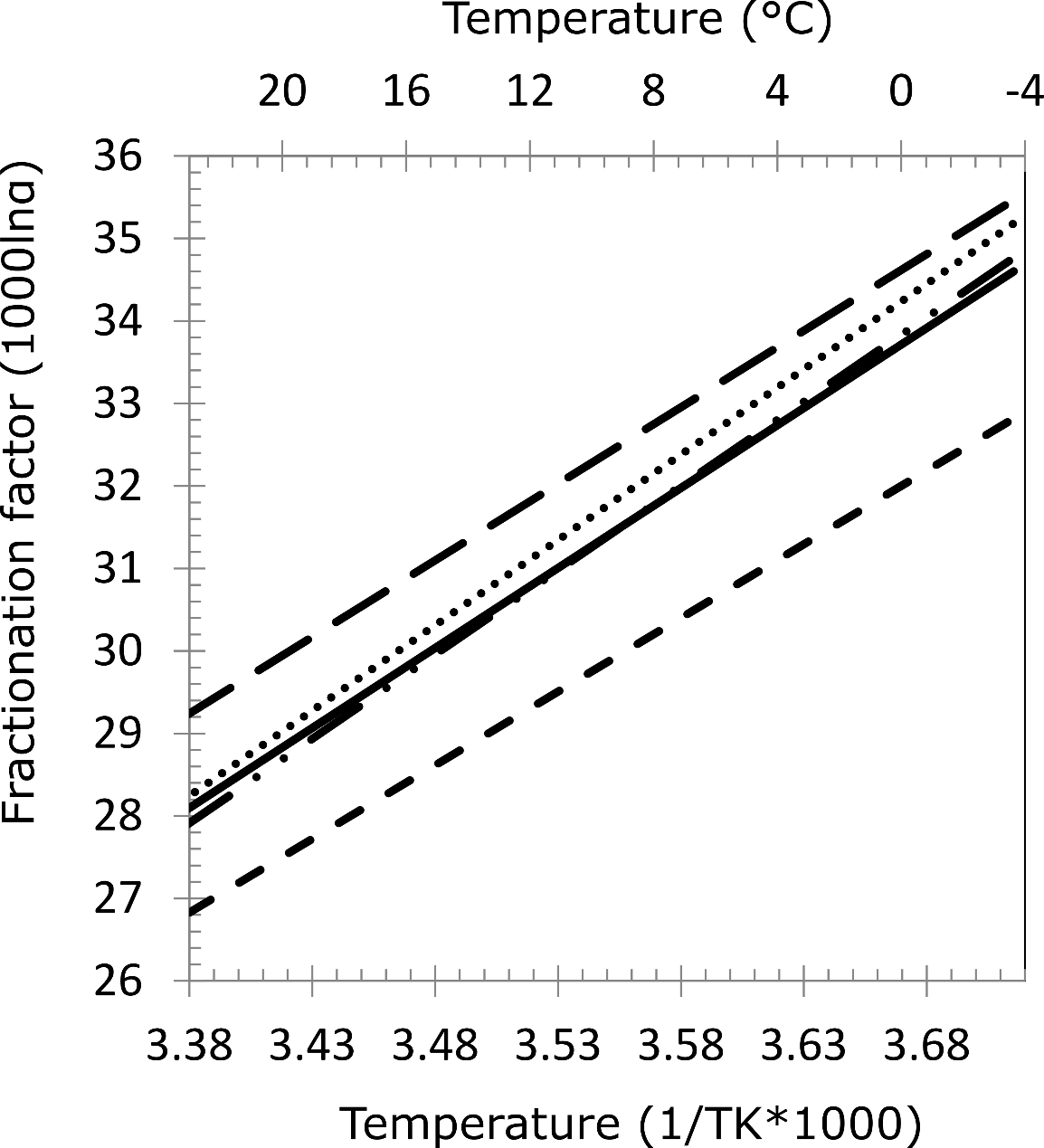
**

**Appendix S2.** Linear relationships describing influence of temperature on isotopic fractionation between fish otolith aragonite and water (1000lnα) from earlier published studies of Patterson *et al.* (1993) (uppermost dashed line), Storm-Suke *et al.* (2007) (dotted line), Godiksen *et al.* (2010) (dashed-dotted line) and Hanson *et al.* (2013) (lowermost dashed line). Solid line represents the average of four presented models used in this study.

**Appendix S3.** Main salmon prey (sprat, herring and three-spined stickleback) mean *δ*^13^C values, standard deviations (sd) of *δ*^13^C values, and sampling numbers (n) in each of their sampling locations used in the study.

| δ^13^C mean | sd | n | Latitude | Longitude |
| --- | --- | --- | --- | --- |
| -17.22 | 0.74 | 5^1^ | 55.0000 | 13.0000 |
| -15.42 | 0.69 | 5^1^ | 58.7500 | 11.0000 |
| -17.96 | 0.71 | 5^1^ | 56.0000 | 15.5000 |
| -19.38 | 0.53 | 2^2^ | 59.4462 | 23.3668 |
| -20.32 | 0.64 | 2^2^ | 59.5607 | 25.3188 |
| -20.73 | 0.64 | 5^2^ | 59.7500 | 21.5000 |
| -20.82 | 0.82 | 97^2^ | 59.7500 | 23.5000 |
| -20.93 |  | 1^2^ | 59.7500 | 25.5000 |
| -21.2 |  | 1^2^ | 60.1134 | 19.0857 |
| -19.56 | 0.71 | 2^2^ | 60.1610 | 26.5568 |
| -21.47 | 0.89 | 109^2^ | 60.2500 | 21.5000 |
| -20.55 | 1.24 | 90^2^ | 60.2500 | 26.5000 |
| -22.1 |  | 1^2^ | 60.7500 | 17.5000 |
| -21.2 |  | 1^2^ | 60.7500 | 19.5000 |
| -21.59 | 0.76 | 81^2^ | 60.7500 | 20.5000 |
| -22.7 |  | 1^2^ | 60.7500 | 21.5000 |
| -21.1 |  | 1^2^ | 61.2500 | 18.5000 |
| -21.54 | 1.05 | 2^2^ | 61.2500 | 19.5000 |
| -21.3 |  | 1^2^ | 61.2500 | 20.5000 |
| -21.82 | 0.71 | 62^2^ | 61.7500 | 20.5000 |
| -21.1 |  | 1^2^ | 61.7500 | 21.5000 |
| -21.25 | 0.59 | 10^2^ | 62.2500 | 18.5000 |
| -21.95 | 0.75 | 6^2^ | 62.2500 | 20.5000 |
| -22.42 | 0.05 | 3^2^ | 62.7500 | 20.5000 |
| -22 | 0.63 | 11^2^ | 63.2500 | 20.5000 |
| -24.2 | 0.61 | 45^3^ | 64.7500 | 24.2500 |

^1^Values from Angerbjörn *et al.* 2006; ^2^Kiljunen *et al.,* 2008; ^3^Sinisalo *et al.,* 2006.

References

Angerbjörn, A, Börjesson, P. & Brandberg, K. (2006) Stable isotope analysis of harbour porpoises and their prey from the Baltic and Kattegat/Skagerrak Seas. *Marine Biology Research*, 2, 411–419.

Kiljunen, M., Peltonen, H., Jones, R. I., Kiviranta, H., Vuorinen, P. J., Verta, M. & Karjalainen, J. /(2008) Coupling stable isotopes with bioenergetics to evaluate sources of variation in organochlorine concentrations in Baltic salmon (Salmo salar). *Canadian Journal of Fisheries and Aquatic Science*, 65, 2114–2126.

Sinisalo, T., Valtonen, E.T., Helle, E. & Jones, R.I. (2006) Combining stable isotope and intestinal parasite information to evaluate dietary differences between individual ringed seals (Phoca hispida botnica). *Canadian Journal of Zoology*, 84, 823–831.
